# Comparison of iguratimod and conventional cyclophosphamide with sequential azathioprine as treatment of active lupus nephritis: study protocol for a multi-center, randomized, controlled clinical trial (iGeLU study)

**DOI:** 10.1186/s13063-021-05475-3

**Published:** 2021-08-11

**Authors:** Qingran Yan, Fang Du, Yuening Kang, Ping Ye, Xiaodong Wang, Jianhua Xu, Jianping Tang, Niansong Wang, Gengru Jiang, Zhijun Li, Xuan Wang, Qin Xue, Xinfang Huang, Xiaoyan Zhang, Ying Zhou, Min Dai, Chunde Bao

**Affiliations:** 1 Department of Rheumatology, Renji Hospital, School of Medicine, Shanghai Jiaotong University, Shanghai, 200001 China; 2 Department of Rheumatology, The First Affiliated Hospital of Anhui Medical University, Hefei, 230022 China; 3 Department of Rheumatology and Immunology, Tongji Hospital, Tongji University School of Medicine, Shanghai, 200065 China; 4 Department of Nephrology, Shanghai Jiao Tong University Affiliated Sixth People’s Hospital, Shanghai, China; 5 Renal Division, Department of Internal Medicine, Xin Hua Hospital Affiliated to Shanghai Jiao Tong University School of Medicine, Shanghai, 200092 China; 6 Department of Rheumatology and Immunology, The First Affiliated Hospital of Bengbu Medical College, Bengbu, 233004 Anhui China; 7 Department of Rheumatology, Shanghai East Hospital, School of Medicine, Tongji University, Shanghai, 200120 China

**Keywords:** Systemic lupus erythematosus, Active lupus nephritis, Randomized clinical trial, Iguratimod, Cyclophosphamide, treatment

## Abstract

**Background:**

Systemic lupus erythematosus (SLE) is an autoimmune disease that can involve multiple organs or systems. Lupus nephritis (LN) is associated with high mortality and morbidity. However, plenty of patients do not respond to present treatment or relapse. Iguratimod (IGU) is a new small molecular, anti-rheumatic drug and has shown the potential for drug repurposing from rheumatoid arthritis (RA) to LN treatment. It has been approved for treating RA in northeast Asia. Beyond expectation in a recent observational study, over 90% of thirteen refractory LN patients responded to iguratimod monotherapy in 24 weeks, with no steroids dose increasing or any other medication add-on during the entire follow-up.

**Methods/design:**

This study is a multi-center, randomized, 52-week parallel positive drug-controlled study. The study was designed as a head-to-head comparison between the iguratimod and present first-line therapy on LN patients. A total of 120 patients (60 patients each group) is in the enrolling plan. All enrolled patients are assigned randomly into trial and control groups. The patients will be selected from six study sites in China and will all have biopsy-proven active lupus nephritis. In the first 24 weeks of the trial, IGU is compared with cyclophosphamide as an induction therapy, and in the second 24 weeks, IGU is compared with azathioprine as a maintenance therapy. The primary outcome is renal remission rate including both complete remission and partial remission at week 52, which will be analyzed using a non-inferiority hypothesis test.

**Discussion:**

Most patients diagnosed with SLE will develop LN within 5 years and LN remains a major cause of morbidity and death for SLE patients. Although some medications are proven effective for the treatment of this condition, at least 20–35% LN patients have to suffer from relapse or ineffective treatment and medication intolerance is also frequent. This trial is designed to demonstrate whether iguratimod can be used as an alternative induction or maintenance therapy in subjects who have lupus nephritis. Data from this study will provide an evidence on whether or not iguratimod should be recommended to active LN patients.

**Trial registration:**

ClinicalTrials.govNCT 02936375. Registered on October 18, 2016.

**Supplementary Information:**

The online version contains supplementary material available at 10.1186/s13063-021-05475-3.

## Introduction

Systemic lupus erythematosus (SLE) is an autoimmune disease that can involve multiple organs or systems [1–3]. Lupus nephritis (LN) is associated with high mortality and morbidity rates. Over recent decades, substantial progress has been made in developing immunosuppressant agents and biologic therapies [4]. However, a significant proportion of patients either do not respond to first-line immunosuppressive drugs or quickly relapse after initial remission. Approximately 5-20% of patients especially in class IV LN (44% of patients) will experience continued worsening of renal function and go on to develop end-stage renal disease in developing vs developed countries [5, 6].

To treat LN, a high dose of steroids plus traditional immunosuppressants, especially cyclophosphamide (CYC) or mycophenolate mofetil (MMF), is still the first-line option in most clinical recommendations [4, 7]. In addition, B cell depletion therapy with rituximab, multiple target therapy with a combination of MMF, and calcineurin inhibitor have emerged as the second-line choice for LN treatment [4]. Of note, these newly recommended regimes, as well as other promising agents that succeeded in recent phase III LN trials, such as belimumab and voclosprorin, are supposed to be applied in combination, for most successful studies on LN adopted add-on strategy. For example, rituximab has been added to another immunosuppressant, typically CYC [8–10], and belimumab and voclosprorin were both added to MMF in phase III trials [11, 12]. The add-on strategy may help to ensure efficacy, but could raise extra safety concerns and may make drug adjustment more difficult when a patient does not respond or tolerate the combinational regime. With this circumstance, a new instead-of medicine for LN would well meet the clinical request.

Iguratimod is a new small molecular, anti-rheumatic drug and has shown the potential for drug repurposing from rheumatoid arthritis (RA) to LN treatment. It has been approved for treating RA in northeast Asia. According to data from RA clinical trials in Japan and China, iguratimod is superior to a placebo and non-inferior to methotrexate and sulfasalazine [13–16]. In a preclinical study on lupus, iguratimod prevented autoimmune nephritis in MRL/lpr mice, decreased the amount of proteinuria, and reduced immune complex deposition [17]. Beyond expectation in a recent observational study, over 90% of thirteen refractory LN patients responded to iguratimod monotherapy in 24 weeks, with no steroids dose increasing or any other medication add-on during the entire follow-up [18].

Previous studies on possible mechanisms have provided the-other-side evidence supporting the rationale for using iguratimod to treat lupus, especially the interference with B cell differentiation. It was found to suppress B cell production of immunoglobulins over a decade ago [19]. In a phase III clinical trial on RA, iguratimod reduced serum immunoglobulin concentrations [14, 16]. In RA and lupus animal models, iguratimod has decreased autoantibody titers, including anti-collagen antibody [20, 21] and anti-double strand (dsDNA) antibody [17]. Interestingly, iguratimod reportedly decreases peripheral plasma cell counts without affecting the total B cell population in MRL/lpr mice [17] and patients with RA who are receiving iguratimod monotherapy [22]. Further investigation has shown that iguratimod regulates the key transcription factors affecting plasma cell differentiation, especially Blimp-1, through the PKC/Egr1 axis [22]. Given the strong evidence of implicating immunomodulatory of iguratimod, we designed and implanted a multi-center randomized, open-labeled, parallel positive drug control clinical trial with non-inferiority hypothesis. This trial focused on patients with active lupus nephritis. And our research is the first randomized controlled trial of iguratimod designed to treat lupus nephritis as well as SLE in the world. We hypothesize that iguratimod monotherapy has non-inferior efficacy to traditional CYC-azathioprine (AZA) sequential treatment on renal remission rate of at week 52 of treatment.

## Methods/design

### Objective

This study is aimed to explore the efficacy and safety of iguratimod for treating biopsy-proven active LN patients, using an instead-of designing. We hypothesize that iguratimod monotherapy has non-inferior efficacy to traditional CYC-azathioprine (AZA) sequential treatment on renal remission rate of at week 52 of treatment.

The secondary objectives include exploring the effect, safety, and tolerance of iguratimod in active LN patients, and recording indicators of immunological markers, laboratory biomarkers, and behavioral factors during the trial.

### Study design

**Fig. 1:**
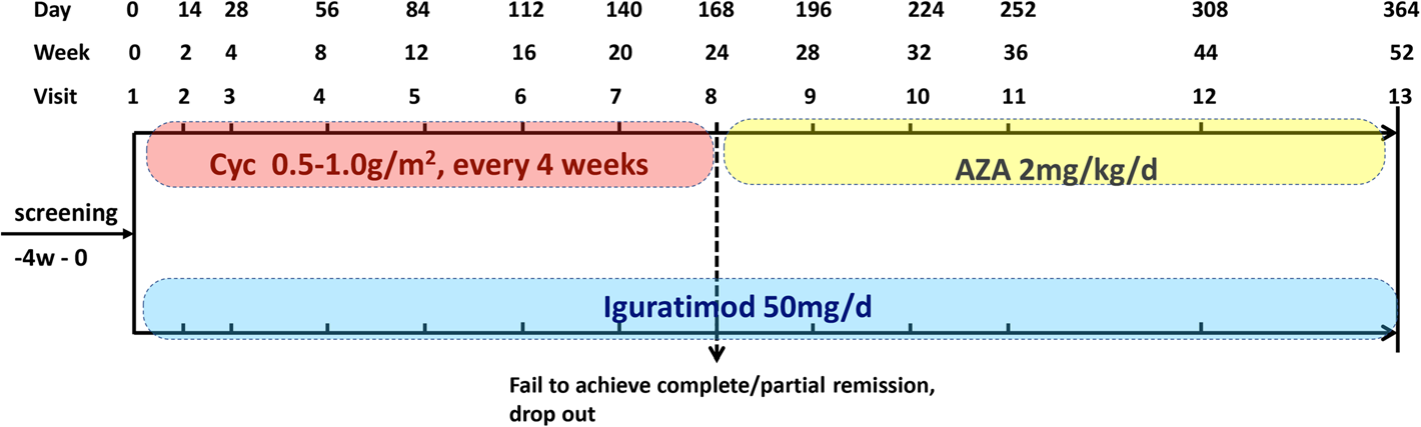
Trial design

**Fig. 2:**
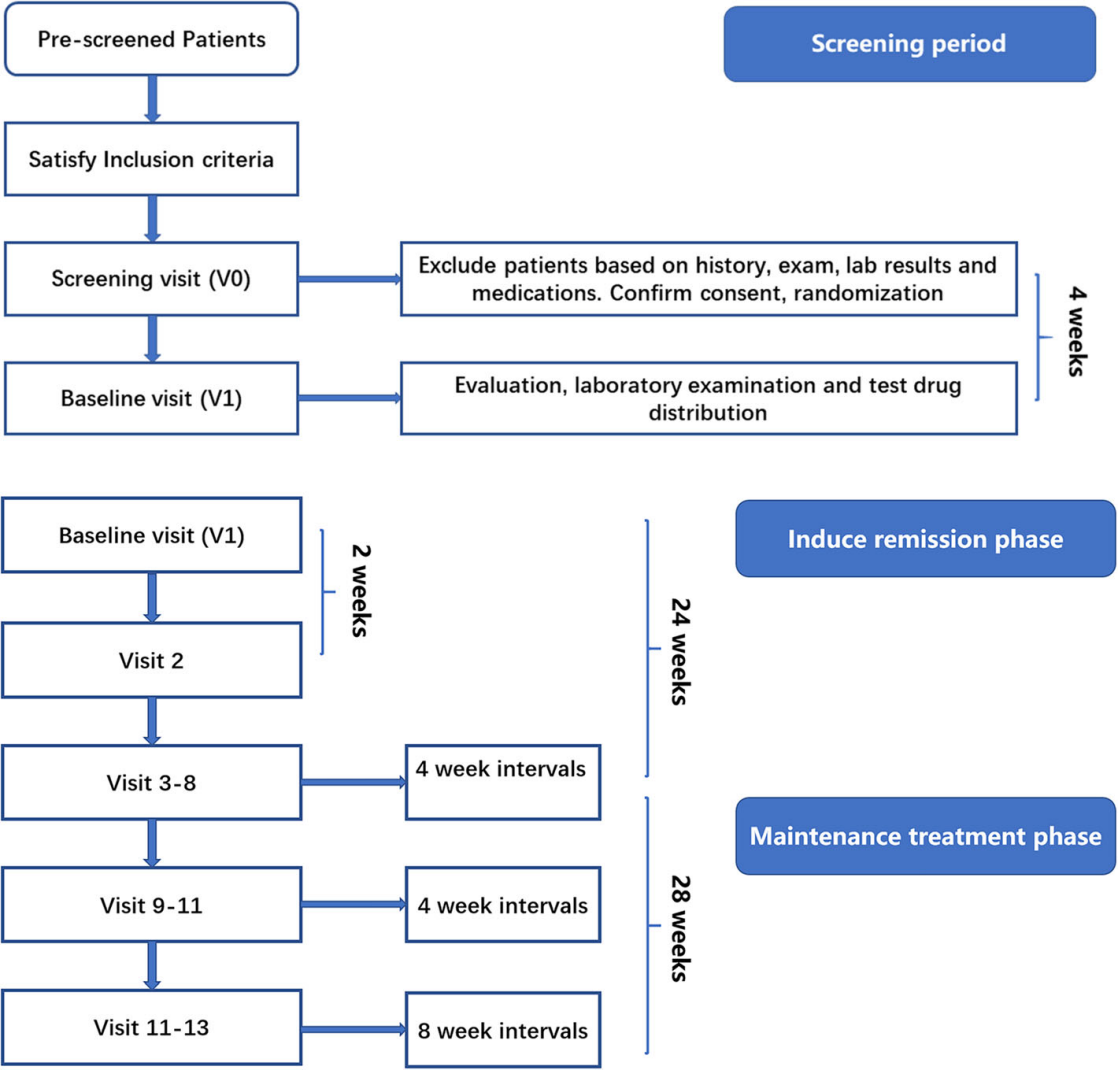
Study flow of iGeLU trial

This study is a multi-center randomized, open-labeled, parallel positive drug control, clinical trial with non-inferiority hypothesis. The patients will be selected from the rheumatology or nephrology department of six study sites after being diagnosed with active LN by biopsy-proved in recent three months and satisfying the inclusion criteria. The flow chart of the study is shown in Figs. 1 and 2. The trial covers the period from August 2017 to August 2023. The Standard Protocol Items: Recommendations for Interventional Trials (SPIRIT) Checklist can be found in Additional file 1.

### Recruitment and consent

A total of 120 subjects with active LN will be recruited in six China academic hospitals: Renji Hospital, Shanghai Jiaotong University School of Medicine; The Sixth People's Hospital Affiliated to Shanghai Jiaotong University School of Medicine; The First Hospital Affiliated to Anhui Medical University; Tongji Hospital Affiliated to Tongji University; Xinhua Hospital Affiliated to Shanghai Jiaotong University School of Medicine; The First Hospital Affiliated to Bengbu Medical College. All six research centers compete for inclusion.

All candidates will receive clinical study information about the trial. Written consent will be obtained from each participant. The purpose, procedures, and potential risks and benefits of the study will also be explained thoroughly to the participants. The participants will be able to withdraw from the study at any time without consequence. Appointments are free of cost to patients. A copy of the signed consent form will be given to the participant and a further copy will remain in the patient’s records at the recruitment site.

### Study procedures

**Fig. 3:**
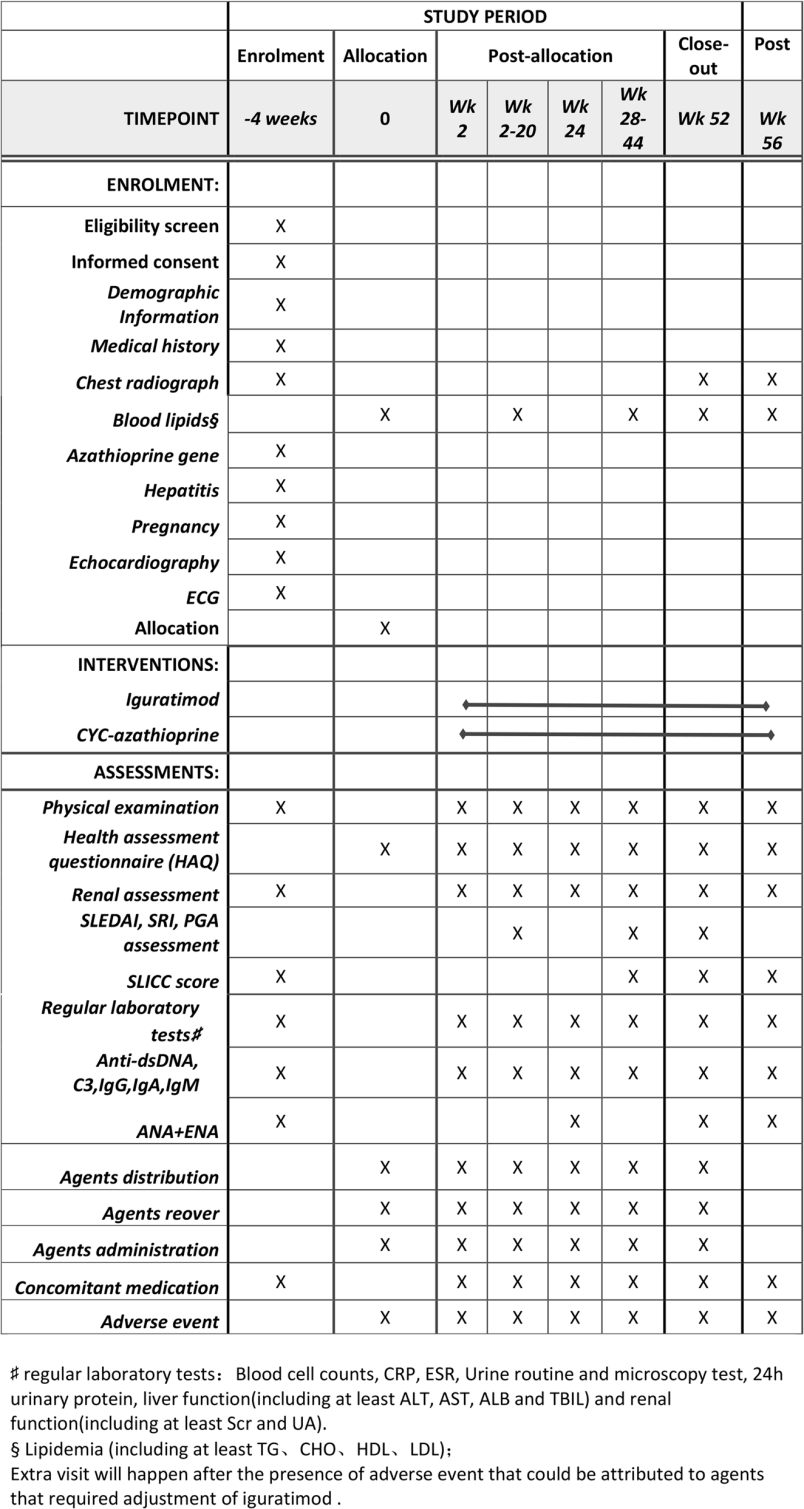
SPIRIT figure of participant timelines

Prescreened patients, who are in the specified age range, have active lupus nephritis, will be scheduled for a screening visit (Figs. 2 and 3). After obtaining written informed consent, the subject will complete medical history, family history, and physical examination. Concomitant medications will be recorded. Laboratory testing will be done, including complete blood count with differential, blood chemistry, urinalysis, urine routine, 24-h urine protein, pregnancy test, C reactive protein, erythrocyte sedimentation rate, anti-dsDNA body, antinuclear antibody (ANA), immunoglobulin, and complements 3 and 4. Individuals who pass the screening laboratory testing and the physical examination will be scheduled for a baseline visit, to take place within 4 weeks of the screening visit.

At the baseline visit (randomization visit), the patient will first complete a physical examination and review of concomitant medications and events will be carried out by the investigator. The following items will be completed: health assessment questionnaire (HAQ), evaluation for lupus, the modified SLEDAI-2 K disease activity instrument for SLE [23], British Isles lupus assessment group score (BILAG score); the SLICC/ACR Damage Index [24] and the provider’s global assessment (PGA) and SLE responder index (SRI) face to face with clinicians.

A random number table generated using Statistical Analysis Software (SAS), version 9.2, using the randomization method, will be used to assign the participants in a ratio of 1:1, with 60 patients each in the trial group and control group. Participants in the iguratimod treatment group will additionally receive 50 mg oral iguratimod. And participants in the control group were treated with pulse cyclophosphamide for 24 weeks and sequential oral azathioprine.

The study participants will be followed for a total of 52 weeks, including the enrolment and follow-up periods (Figs. 1 and 2), After randomization at visit 1, visit 2 will occur at 2-week, visits 3–10 will occur at 4-week intervals, and visits 11–12 will occur at 8-week intervals following the schedule. Each study visit will include medication and adverse event review, physical examination, laboratory examination, completion of the SLEDAI, SLICC criteria, and ACR criteria. Medication compliance is determined by pill count and a new supply of pills dispensed.

Participants who achieve renal remission at the end of 52-week visit will undergo extended follow-up visits. Any potential adverse events or significant changes in the subject’s medical condition will prompt an unscheduled study visit. Unscheduled visits may be performed to document adverse events, worsening of the subject’s medical condition, or withdrawal from the study. Within 4 weeks of completion of the treatment period, participants will undergo another visit to make sure no new adverse events happen.

Standard protocol items on the list of recommendations for interventional trials (SPIRIT) are provided as a completed checklist (Additional file 1).

### Inclusion criteria

Inclusion criteria are shown in Table 1.

**Table 1:** Inclusion criteria of iGeLU study

1	Age 18–65 years, male or female
2	Diagnosed as active lupus nephritis (meet the following three standards at the same time):
	a Participants fulfill the SLE classification criteria by the American College of Rheumatology (ACR) in 2009.
	b Proteinuria of no less than 1.0 g/24 h within the 4 weeks before enrollment, with or without microscopic hematuria.
	c Active lupus nephritis confirmed by biopsy and classified as class III/IV/V type or compound type (III+V or IV+V) according to the classification of lupus nephritis within 3 months prior to screening.
3	Systemic Lupus Erythematosus Disease Activity Index 2000 (SLEDAI-2000) scores ≥ 8 points within 2 weeks before enrollment.
4	Weight no less than 40 kg.
5	The participant agrees to take measures for birth control contraception
6	The patient agrees to participate and provides informed consent

### Exclusion criteria

Exclusion criteria are shown in Table 2.

**Table 2:** Exclusion criteria of iGeLU study

1	Severe manifestations of SLE, including:
	a. Neuropsychiatric lupus within 1 month before screening.
	b. Extensive crescentic glomerulonephritis confirmed by biopsy with a ratio of crescents higher 50%.
	c. Estimated glomerular filtration rate (eGFR) < 60 ml/min/1.73m^2^ (calculated by the EPI formula) at least two tests within 30 days before screening and at least 14 days between the two tests.
	d. Evidence of significant abnormal laboratory value in peripheral blood not related to lupus (white blood cells (WBC) < 3 × 10^9^/L, platelets (PLT) < 50 × 10^9^/L) within 1 week before screening.
	e. Moderate to severe anemia within one week before screening.
	f. AST and ALT values more than two times the upper limit of normal within one week before screening.
2	Participant has been diagnosed with another autoimmune disorder, including but not limited to: rheumatoid arthritis, Sjogren’s syndrome, inflammatory myositis, systemic sclerosis, autoimmune liver disease.
3	Prior use of the following agents within 3 months of screening.
	a. MMF or CNIs for more than 14 days in accumulation.
	b. Anti-TNF-α, anti-interleukin-6 (IL-6), anti-IL1 or JAK inhibitor.
	c. B cell depletion therapy (e.g., anti-CD20 monoclonal antibody) or anti-B lymphocyte stimulator therapy (e.g., belimumab)
	d. High dose of glucocorticoid therapy (> 100 mg/d calculated by prednisone).
	e. Plasma exchange, blood adsorption, hemodialysis, or mesenchymal stem cell translation.
4	Gene test for AZA demonstrates a high risk of side effects.
5	Active bacterial, viral, fungal, or opportunistic infection during the screening period.
6	Active viral hepatitis.
7	Active tuberculosis.
8	Receipt of a live or live-attenuated vaccine within 4 weeks of screening.
9	History of peptic ulcer or upper gastrointestinal hemorrhage, prior use of warfarin or other anticoagulant drugs.
10	Suspected or confirmed history of alcohol or drug abuse within two years
11	History of malignant neoplasm except carcinoma in situ, basal cell carcinoma and cured carcinoma.
12	Severe, progressive, or uncontrolled pulmonary, cardiac, or hypertension medical condition.
13	Participant suspected of mental disability.
14	Participant with epilepsy or nervous system dysfunction.
15	Pregnant, breastfeeding, or unwilling to practice birth control during participation in the study.

### Discontinue criteria

Any participant unwilling or unable to continue the clinical trial for any reason could withdraw informed consent and withdraw the trial. And any subjects who have not reached remission or partial remission by 24 weeks will be terminated.

Termination of study may occur by investigators for the following reasons (Table 3):

**Table 3:** Study of iguratimod in lupus nephritis discontinue criteria

1	Participants experiencing serious adverse reactions during the study
2	Fatal comorbidities happen, including but not limited to severe infection
3	Participants whose condition deteriorates within the first 24 week
4	Participants who fail to achieve remission (CR or PR) until the end of week 24
5	Pregnant
6	Any other circumstances under which the investigator considers the patient to be unable to finish the study

### Interventions

#### Experimental arm

1. Iguratimod (25 mg per tablet, purchased from Simcere Pharmaceutical). Participants in the iguratimod group will be prescribed oral iguratimod at a dose of 25 mg twice daily until the end of trial.

#### Control arm

1. Cyclophosphamide (CYC, 0.2 g per potion). Participants in the control group will be prescribed intravenous CYC as induction immunosuppression at a dosage of 0.5–1.0 g/m^2^ once for 4 weeks.
2. Azathioprine (AZA, 50 mg per tablet). Participants in the control group who has achieved remission (PR/CR) at week 24 will be prescribed oral azathioprine at an initial dose of 50 mg/d. If there is no acute leukocytopenia in weeks 25 to 28, the dosage would be increased to 2 mg/kg per day in the next 1 month.

#### Combination therapy

1. Glucocorticoid: All participants will be treated with glucocorticoid (calculated by prednisone) following the routine:
  1.1 Oral prednisone (1 mg/kg/d) for first 4 weeks.
  1.2 The dosage of prednisone will be reduced by 5–10 mg every 2 weeks from the 5th week until 30 mg/d, followed by reduction of 2.5-5 mg for every 2 weeks, followed by a taper to no more than 10 mg/d until the end week 24, followed by maintenance therapy with daily oral prednisone no more than 10 mg/d.
  1.3 Prednisone could be increased to 1 mg/kg/d when participants experiencing severe extrarenal symptoms for no more than 2 weeks.
2. Anti-malaria drugs: Hydroxychloroquine should be used during the study with no more than 5 mg/kg per day.
3. Antihypertensive drugs: Participants could continue angiotensin-converting enzyme inhibitor/ receptor blocker (ACEI/ARB) if he or she has already received ACEI/ARB therapy for more than one month before screening. It is not allowed to add additional ACEI/ARB drugs or increase dose of ACEI/ARB agents during the study. Other medications could be added based on the conditions of participants to control hypertension such as β-blockers, calcium channel blockers, and diuretics.
4. The other agents permitted to use during the trial are listed as follow:
  4.1 Calcium and active vitamin D agents.
  4.2 Aspirin or other antiplatelet agents.
  4.3 Statins or other lipid-lowering agents.
  4.4 Proton pump inhibitors (PPI) or other gastric mucosal protective agents.
  4.5 Hypoglycemic agents.
  4.6 Nonsteroidal anti-inflammatory drug (NSAIDs) no more than 7 days every time.
  4.7 Other appropriate medications for comorbidities, yet systemic administrations of steroids for other purposes such as asthma is not allowed.
5. All other traditional immunosuppressant agents or target therapy except CYC and AZA, will not be allowed to use during the study, including but not limited to MMF, CNIs, rituximab, and JAK inhibitors.

### Outcomes measures

#### Primary outcome

The primary outcome is reported as the proportion of participants with renal remission (PR or CR) at 52 weeks.

#### Key secondary outcomes

The key secondary outcomes include renal remission rate by week 24, renal flare rate by week 52, number of treatment-related adverse events, and changes in SLEDAI-2 k score, BILAG score, and PGA. The detail of key secondary outcomes is displayed in Table 4.

**Table 4:** Study of iguratimod in lupus nephritis key secondary outcomes

	Key secondary outcomes
1	Proportion of participants with renal remission by 24 weeks.
2	Proportion of participants with flare by 52 weeks
3	Number of participants with treatment-related adverse events which are assessed by CTCAE v4.0b by 52 weeks
4	SLE disease activity index (2000) (SLEDAI-2 K) score by 52 weeks
5	British Isles lupus assessment group (BILAG) score by 52 weeks
6	Patient general assessment (PGA)

#### Exploratory secondary outcomes

Exploratory secondary outcomes are also planned as follows (Table 5):

**Table 5:** Exploratory secondary outcomes

	Exploratory secondary outcomes
1	Proportion of participants with complete renal remission by 52 weeks
2	Time to renal remission.
3	Duration of remission.
4	Duration of flare.
5	24-h urinary protein levels
6	Proportion of participants with double serum creatinine level and time to double serum creatinine.
7	Proportion of participants with end-stage renal disease (ESRD) and time to ESRD.
8	Proportion of participants with extrarenal flare.
9	Proportion of participants with remission defined by SLE responder index (SRI) by 24 weeks and 52 weeks.
10	Proportion of participants with positive anti-dsDNA or antibody titer.
11	Complement 3 and complement 4 levels.
12	Serum Immunoglobulin levels.

### Assessment criteria

#### Renal assessment criteria

1. Renal remission
  - Complete remission (satisfying all criteria):
    24 h urine protein < 300 mg.
    Normal counts of urine blood cells or casts (RBC< 5/HP, WBC< 5/HP).
    Normal serum albumin level.
    Normal serum creatinine and creatinine clearance (CCr).
  - Partial remission (satisfying all criteria):
    24-h urine protein between 300 mg and 2000 mg with at least a 50% decrease from the baseline.
    Serum albumin concentration over 30 g/L
    Serum creatinine and creatinine clearance levels remain stable during trial, or increase/decrease no more than 25% from the baseline.
  - No response (satisfying any of the following criteria):
    24 h urine protein > 1 g
    24 h urine protein ≤ 1 g but decrease no more than 50% from the baseline.
    Serum albumin < 30 g/L
    Abnormal creatinine levels at enrollment cannot return normal after treatment.
2. Renal flare
  - For participants with 24 h urine protein between 500 and 2000 mg at baseline achieving remission, reproducible increase of 24 h urine protein by ≥ 50% with or without active urine sediment and cannot recover removing the inducement within 2 weeks.
  - For participants with 24-h urine protein below 500 mg at baseline achieving remission, reproducible increase of 24 h urine protein over 1 g with or without active urine sediment and cannot recover removing the inducement within 2 weeks.
  - Reproducible increase of serum creatinine by ≥ 50% and cannot recover removing the inducement within 2 weeks.

#### Efficacy evaluation criteria

1. SLE responder index (SRI) assessment (satisfying all criteria):
  - Systemic Lupus Erythematosus Disease Activity Index (2000) (SLEDAI-2 K) scores improve ≥ 4 points from baseline.
  - No deterioration in the provider’s global assessment (PGA) scores (scores improve < 0.30 points from baseline).
  - British Isles Lupus Assessment Group (BILAG-2004): No A score due to items which are “new” and no more than two B score due to items.
2. Extrarenal symptom assessment: extrarenal flare defined by more than 10 items in Systemic Lupus Erythematosus Disease Activity Index (SLEDAI) which is except abnormal urine test items.

### Data collection and management

Eligible patients who sign written informed consent will be randomly assigned across study centers. The study will be conducted in accordance with the currently approved protocol, International Conference on Harmonisation Good Clinical Practice (ICH GCP), and relevant regulations.

To maintain confidentiality, all laboratory specimens, evaluation forms, reports, and other records will be identified by a coded number and initials only. Case report forms (CRFs), medical reports, and laboratory test reports will make up the main data source file for the subjects in this clinical trial. Investigators must fill out the case report form truthfully. CRF-related data are provided by the research team to a professional institution for clinical data management and study data statistics. Data will be modified by investigators strictly following inductions of case report form with signature and date every time. The collected data in CRF will be audited and verified by referring to the source document by the clinical researchers of the project during a scheduled visit to ensure data integrity with signature and date for every visit.

The medical record data will be entered into the database and consistent with CRFs. After data are input into electronic care report tables, quality control and data audit procedures will be performed by individual investigator to ensure the accuracy and reliability of these data.

### Data monitoring

During the study, an independent data monitoring committee (DMC) will be set up to carry out periodic interim evaluation and optimize the study when appropriate based on the results of the interim evaluation. The DMC is authorized to discontinue the clinical study in case of unexpected adverse reactions. During implementation of the project, staff of DMC will check each electronic case report form (eCRF) for validity and consistency periodically or irregularly, and study compliance will be checked, so that data integrity and accuracy will be fully guaranteed and authenticity and reliability of the study results are ensured.

### Sample size calculation

The effect of iguratimod was carried out as one of the main efficacy indexes, the primary hypothesis is that iguratimod therapy is not inferior to CYZ+AZA therapy.

Thus, the required sample size is:

$$ {n}_A=\kappa {n}_B\ \mathrm{and}\ {n}_B=\left(\frac{p_A\left(1-{p}_A\right)}{\kappa }+{p}_B\left(1-{p}_B\right)\right){\left(\frac{z_{1-a}+{z}_{1-\beta }}{p_A-{p}_B-\delta}\right)}^2 $$

where *p*_*A*_ is the remission rate on the basis of previous investigational study estimated as 0.8, p_B_ is estimated as 0.7 based on related data.

The *δ* value between the two groups is expected to be − 0.1 with a significance level of *α* = 0.05 and an assurance of 1 – *β* = 0.8. Thus, the total sample size was calculated as 53 cases if the iguratimod group and the control group were included at a ratio of 1:1. We will increase the sample size by approximately 10% to reach 60 cases in consideration of the possibility that some patients could miss visits due to the long illness course and follow-up time.

### Statistical analysis

#### Statistical analysis group

The data obtained from the participants of this clinical trial will mostly be divided into a FAS (full analysis set) group, a PPS (per protocol set) group, and a SS (safety set) group.

##### Full analysis set (FAS)

Based on the principle of intentionality treatment (ITT principle), the FAS group will include all randomized subjects who met entry criteria. The primary efficacy analyses will be based on the FAS group, as well as key secondary efficacy and exploratory secondary outcomes. For the FAS group, if a defect value happens at any point or a dropout occurs before the clinical trial ends, the most recent data will be analyzed as if it were obtained at that point in time LOCF (Last Observation Carried Forward Analysis). In a LOCF analysis, a missing follow-up visit value is replaced by (imputed as) that subject’s previously observed value, that is, the last observation is carried forward. And patients who did not reach remission would be identified as ineffective subjects.

##### Per protocol set modify (PPS/PPSM)

The PPS group will include participants from the FAS group who successfully completed this clinical trial according to the trial plan. Participants in PPS group who seriously violate trial protocol or take a forbidden drug or do not obey a schedule can be excluded. ITTM analysis set and the PPSM set will be applied to sensitivity analysis to check for overall robustness of the primary analyses.

##### Safety set (SS)

The safety set group will include participants who took the iguratimod and underwent safety assessment at least once.

#### Statistical analysis method

Per protocol set will pick up from the full analysis set for analysis. Statistical analysis of the efficacy of the study will be performed using statistical data sets that meet the protocol. We will calculate the average, standard deviation, minimum value and maximum value for continuous data, and we will calculate the frequency and percentage for categorical data.

For the purpose of group comparisons, we will compare the demographic data for the two groups by using the *χ*^2^ test, Fisher’s exact test, *t* test, non-parametric statistical method, or CMH analysis.

Efficacy analysis will be performed to compare the efficacy of each group. Continuous variables will be compared by using *t* test and covariance analysis model considering central effect (two sides: processing factor and baseline factor). Data of steroid reduction will be compared using survival analysis, and Kaplan–Meier curve method will be used for survival analysis.

Descriptive statistics (presented in table form) will be used for safety and tolerability data. If necessary, Fisher’s exact test will be conducted for the percentage of adverse events between the two groups. Laboratory test results will be used to describe conditions when abnormal results happen and the connection with the trial agents.

A two-sided test with a 0.05 significance level will be applied, *P* values ≤ 0.05 will be considered statistically significant. All statistical analyses will be performed with SAS software, version 9.13 (SAS Institute, Cary, NC, USA). The treatment arms will be masked to both outcome assessors and data analysts and maintained according to standard practices.

### Safety assessments

#### Safety analysis

Subjects will suffer from side effects of drugs and treatment failure during the trial. Adverse events reported in clinical trials of iguratimod for rheumatoid arthritis patients are as follows:

The most common adverse effect is impaired liver function. Common adverse effects include leukopenia, stomach discomfort, nausea, bloating, stomachache, anorexia, rash, nausea, abdominal distension, thrombocytopenia, sour regurgitation, abdominal pain, blurred vision, skin itching, duodenitis, gastritis, fecal occult blood, hair loss, insomnia, abnormal electrocardiogram, menstrual disorders, and anemia. Rare adverse effects include diarrhea, dyspepsia, belch, gastric ulcer, reflux esophagitis, duodenal ulcer, gastric antrum bleeding, vomiting, fever, cough, dry mouth, Oral ulcers, facial edema, skin edema, fatigue, chest tightness, chest pain, positive urine protein, elevated total bilirubin, flu-like symptoms, upper respiratory tract infection, and acne-like gastritis. Most of the adverse effects mentioned above will alleviate or disappear spontaneously after withdrawal. It is possible that new adverse events occur during the trial.

The research team will assess all participants at the end of 24 weeks. Participants will withdraw from the trial and switch to another therapy if they fail to achieve remission. The research team will advise early termination of the trial in the event of safety concerns or lack of any treatment effect.

All AEs will be recorded at trial visits for the 52 weeks of by the member of the research team and filled in in the case report form. All adverse events must be judged for their character, severity, and potential relationship to the study treatment. All AEs will be judged by a medically qualified member of the research team or the sponsor will be followed until resolution or the event is considered stable, clinically insignificant, or asymptomatic. All related AEs that result in a participant’s withdrawal from the study or are present at the end of the study should be followed up until a satisfactory resolution occurs.

### Adverse events assessment and management

#### Adverse events definition

1. Adverse event definition: An adverse event is defined as any untoward medical occurrence (including deterioration of a pre-existing medical condition) in a patient who has been administered a medicinal product; the event does not necessarily have a causal relationship with this product. Adverse events including but not limited to as follows:
  - Abnormalities in laboratory tests
  - Clinical significant symptoms and signs
  - Overdose
  - Self-discontinuation
  - Drug abuse
  - Drug misuse
  - Drug addiction;
  - Pregnancy events
2. Severe adverse event: It refers to adverse events containing any of the following conditions with nothing about dosage.
  - Death.
  - Lethal events.
  - Participants whose condition deteriorates during the study leading to hospitalization or extension of the original hospital stay.
  - Leading to permanent or severe disability/incapacity
  - Leading to congenital malformations/birth defects

#### Adverse events assessment

The correlation between adverse events and study treatment is divided into five levels: definitely related, probably related, possibly related, possibly unrelated, and definitely unrelated.

The adverse reactions are divided into 3 levels:

- Classification I: Mild (+), participants can continue trial agents without special treatment.
- Classification II: Moderate (++), participants will continue agents after the break or other treatments.
- Classification III: Severe (+++), participants need to suspend medications.

#### Adverse event management

The principles for adverse events are as follows:

1. Abnormalities in blood cell counts or liver function tests

**Table:** 

	Continue routine	Reduction	Withdrawl
Blood cell counts	WBC ≥3× 10^9^/L		WBC < 3× 10^9^/L
Liver function	Transaminase < 1.5 times	1.5 times ≤ transaminase < 3 times	Transaminase level rise ≥ 3 times

Hepatoprotective drugs and whitening drugs can be added as appropriate for the above conditions. The research team will adjust the medication regimen according to the relevance of the adverse event and the trial agents. Participants will be prescribed trial agents with the original dose. The treatment can be repeated 3 times, and agents will not be added to the original dosage for the fourth time.

2. Participants will withdraw from the trial when they are allergic to agents and treated according to the clinician’s experience
3. Other adverse events: Participants will be diagnosed and treated following related routine

### Ethical considerations

The present study is being conducted in accordance with the Declaration of Helsinki, Quality management standard of drug clinical trials (GCP), and relevant clinical study research regulations in China. The protocol was approved by the Ethics Committee of the Renji Hospital, Shanghai, China.

## Discussion

Systemic lupus erythematosus (SLE) is an autoimmune disease of unknown cause involving various tissues and organs damaged, and lupus nephritis (LN) is one of the most severe organ manifestations of SLE. Most patients diagnosed with SLE will develop LN within 5 years [25–27]. Some patients will progress to end-stage renal disease, LN remains a major cause of morbidity and death [27–29]. The main goal of treatment is to palliate symptoms, defer progression, reduce disease progression-related complications and mortality rate, and also preserve fertility for female patients. Glucocorticoids combined with either cyclophosphamide or MMF currently is used as first-line agents. On top of this, there are some other medications recommended such as calcineurin inhibitors, leflunomide, and other biological agents [30]. However, some patients still suffer from relapse or ineffective treatment and medication intolerance is also frequent. Therefore, the demand for new drugs continues.

IGU is a novel small-molecule immunomodulatory agent. IGU can effectively inhibit expression of various inflammatory factors, inhibit B cells from producing immunoglobulins and autoantibodies, downregulate T cell-mediated cellular immunity, accelerate bone formation, and exert some activity against anti-pulmonary fibrosis. IGU is widely used in the treatment of RA; both monotherapy and combination therapy suggest good efficacy and safety. In addition, the clinical studies suggest that IGU has a good effect on other rheumatic diseases, such as Sjögren’s syndrome, ankylosing spondylitis, and IgG4-related diseases [31].

Our study was designed as a head-to-head comparison between iguratimod with a classic LN therapy, CYC ensued with AZA. In the first 24 weeks of the trial, IGU is compared with cyclophosphamide as an induction therapy, and in the second 24 weeks, IGU is compared with azathioprine as a maintenance therapy. In the previous observatory LN study, over 90% refractory LN patients responded to iguratimod monotherapy without any steroids increasing in the first 24 weeks of treatment [18]. Given to this outstanding effectiveness of IGU, the result of this study is expectable and promising.

The availability and use of an unproven, but relatively safe, drug for people with LN and the reluctance of potential subjects are the major obstacles. Also, it might be a hindrance when we persuade patients to underwent kidney biopsy. A limitation of this study is that the longtime effect of the agent or therapy was unable to confirm due to the limited study duration. However, participants who achieve renal remission at the end of 52-week visit will undergo extended follow-up visits (not reflected in the data).

The iGeLU trial is designed to demonstrate whether iguratimod can induce and maintain remission thereby slowing the progression of disease in subjects who have lupus nephritis. Data from this study will provide an evidence on whether or not iguratimod should be recommended to all lupus nephritis patients, and those patients will be able to make an informed choice about the risks and benefits of this medication.

## Trial status

The current iGeLU study protocol version is 2.1 dated 7 July 2017. Enrollment was initiated in August 2017. The outbreak of COVID-19 forced us to change the protocol and delayed the progress of our trial. Igutimod is used as an induction therapy for lupus nephritis for the first time, which decreases enthusiasm on the patient side, and also caused difficulties in recruiting. Therefore, enrollment completion is anticipated for November 2022. And the estimated study completion date is November 2023.

## Supplementary Information

**Additional file 1.**

## Data Availability

The clinical study report of this trial would be published with the research paper as an appendix. The raw data would be accessible for all the investigators.
